# Expression of Proteinase-Activated Receptor 2 During Colon Volvulus in the Horse

**DOI:** 10.3389/fvets.2020.589367

**Published:** 2020-11-27

**Authors:** Carlotta Lambertini, Augusta Zannoni, Noemi Romagnoli, Cristiano Bombardi, Maria Morini, Francesco Dondi, Chiara Bernardini, Monica Forni, Riccardo Rinnovati, Alessandro Spadari

**Affiliations:** Department of Veterinary Medical Sciences, University of Bologna, Bologna, Italy

**Keywords:** colic, colon volvolus, cytokines, equine, intestine, proteinase-activated receptor 2

## Abstract

Large colon volvulus in horses is associated with a poor prognosis, especially when ischemic-reperfusion injury of the affected intestinal tract develops. Proteinase-activated receptor 2 (PAR_2_) plays an important role in the pathogenesis of inflammation in the gastrointestinal tract. The aim of this study was to evaluate the distribution and expression of PAR_2_ in colonic pelvic flexure of horses spontaneously affected by large colon volvulus (CVH group). Eight horses admitted for severe abdominal colon volvolus and which underwent surgery were included. Colon samples were collected after enterotomy. Data previously obtained from healthy horses were used as a control group. Histologic evaluation was carried out to grade the severity of the colon lesions. Immunofluorescence, western blot and quantitative polymerase chain reaction (RT-qPCR) were carried out on colon samples to evaluate PAR_2_ expression. In addition, the transcriptional profile of cytokines and chemokines was evaluated using RT^2^ Profiler™ PCR Array Horse Cytokines & Chemokines. Three out of the eight patients were euthanised due to clinical deterioration. Immunostaining for PAR^2^ was observed in the enterocytes, intestinal glands and neurons of the submucosal and myenteric plexi. In the CVH horses, the expression of PAR_2_ mesenger RNA (mRNA) did not differ significantly from that of the healthy animals; western blots of the mucosa of the colon tracts showed a clear band of the expected molecular weight for PAR_2_ (~44 kDa) and a band smaller than the expected molecular weight for PAR_2_ (25kDa), suggesting its activation. The gene expressions for C-X-C motif ligand 1 (CXCL1); interleukin 8 (IL8), macrophage inflammatory protein 2 beta (MIP-2BETA) were upregulated in the colic horses as compared with the colons of the healthy horses. Therefore, in the present study, the expression and activation of PAR_2_ in the colons of horses in the presence of an inflammatory reaction like that occurring in those with spontaneous colon volvulus was confirmed.

## Introduction

Colic is the most frequent cause of death in horses, and large colon displacement and volvulus account for 10-20% of horses presented for colic (1). Weight of the large colon, its length, and its lack of extensive mesenteric attachments likely play a role in the development of malpositions (2).

The prognosis is often poor for ischaemic diseases of the colon, such as large colon volvulus and colonic infarction, despite aggressive surgical and medical interventions. The short-term survival rate for horses with large colon volvulus is high, ranging from 35 to 86% while the long-term survival rate decreases to ~30% (1, 3).

The onset time of volvulus, or strangulation of the colon, heart rate and packed cell volume (PCV) prior to admission were associated with patient survival to discharge. Moreover, an increase in heart rate in the postoperative period was associated with a poor outcome. A frequent cause of increase in heart rate is endotoxemia due to cellular damage of the mucosa, associated with reperfusion injury after the surgical reposition of the colon. At the cellular level, reperfusion of the ischemic tissue results in the rapid generation of oxygen-derived free radicals which overwhelms endogenous protective antioxidants and induces cell injury, particularly endothelial injury. Moreover, during reperfusion, the generation of reactive oxygen radicals, the production of proinflammatory enzymes, and the expression of adhesion molecules initiate an inflammatory response which enhances the release of proteinases generated by the immune cells (neutrophils, mast cells) (4) by means of the activation of the coagulation cascade (5).

Proteinase-activated receptor 2 (PAR_2_), a member of a family of G protein-coupled receptors, is largely distributed in the bowel tract, and it is activated by proteinases (such as trypsin and mast cell tryptase). These proteinases are particularly abundant in the guts of patients with inflammatory bowel disease (IBD) and in animal models with induced intestinal ischemia; therefore, the receptor is considered to play an important role in the pathogenesis of inflammation and related chronic pain in the gastrointestinal tract (6–8).

In the intestine, PAR_2_ signaling has been reported to both mediate physiological functions, e.g., ion transport, regulation of the intestinal barrier function and gut motility (9–12), and to exacerbate pathological conditions, such as tumor development and the progression of IBD or food sensitivities (8, 13–16). Due to the number of proteases involved and the multiple cell types affected, the selective regulation of intestinal PARs represents an interesting therapeutic strategy. The expression of PAR_2_ in the equine small intestine after epiploic foramen herniation has recently been described. The authors reported that PAR_2_ is strongly activated in the ischemic small intestine, internalized and progressively degraded in the bowel of horses with a diagnosis of an epiploic foramen hernia (17), confirming the key role of PAR_2_ in this spontaneous pathological model in the small intestine. The authors hypothesized that PAR_2_ could also play a role in the pathogenesis of colonic ischemic changes secondary to volvulus and treated with a surgical procedure.

The aim of this study was to evaluate the distribution and expression of PAR_2_ in the colonic pelvic flexure of horses spontaneously affected by large colon volvulus and to relate their expression to the presence of an inflammatory process.

## Materials and Methods

The study was approved by the Ethical Scientific Committee for Experimental Animals of the University of Bologna (Prot. n 15-IX/, 08/ 05/2012).

### Animals

Horses admitted to the Veterinary University Hospital (VUH) of the University of Bologna for severe abdominal pain and colon volvolus [colon volvulus horse (CVH) group], diagnosed during clinical evaluation at the VUH were recruited for the study.

In detail, the inclusion criteria were: >2 and <20 years of age, no sex distinction, presentation within 6 h of the onset of clinical signs of abdominal pain and a diagnosis of colon volvolus confirmed at exploratory laparotomy. Moreover, surgical inclusion criteria were applied: colon volvulus with a torsion >270° were included; horses with a diagnosis of nephrosplenic entrapment were excluded. To include a uniform population of horses having an ischemic process coming from a clinical recruitment, any horse in which the diagnosis was delayed at the VUH was excluded.

All patients underwent an exploratory laparotomy, and a tract of ~3 × 2 cm of pelvic flexure was resected by an experienced surgeon (AS) during a colotomy for the emptying of the left colon. The horse owners gave informed consent for inclusion in the study and committed to strictly following the rules of the experimental design.

Moreover, there was a control group composed of samples of pelvic flexure collected from eight healthy horses (HLT group) at the slaughterhouse, as previously described (18). Briefly, the horses were considered to be healthy based on clinicopathological and histological intestinal evaluation; there were five females and three geldings, of different breeds having a median age of 10.5 years (range 2–20).

### Sample Collection

The blood samples undergoing clinicopathological evaluation were collected within 30 min from admission of the horses to the clinic. Tubes with K_3_EDTA anticoagulant, citrate and a clot activator were used. The blood samples were processed and analyzed within 1 h from collection, or were stored at −80°C.

A full thickness antimesenteric specimen from the pelvic flexure was taken immediately after the surgical evacuative colotomy. The specimen was dissected and separated into three small symmetric samples in a separate specific laboratory. After several washings with phosphate buffered saline (PBS, Gibco-Invitrogen, Paisley, UK), the intestinal mucosa from the first bioptic sample was scraped using two glass slides. All of the mucosa samples were then frozen in liquid nitrogen and stored at −80°C until RNA and protein extraction. The remaining two full-thickness samples were rinsed in PBS and immediately placed in 10% neutral buffered formalin (for histology) and 4% paraformaldehyde (for immunohistochemistry).

### Clinicopathological Evaluation

All the cases included had a complete blood count (CBC) (ADVIA 2120, Siemens Healthcare Diagnostics, Tarrytown NY, USA) carried out, including haematocrit value (HCT), hemoglobin concentration, erythrocyte indices, platelet count, white blood cell (WBC) with differential WBC counts and a blood smear examination. A chemistry profile, including aspartate transaminase, lactate dehydrogenase (LDH), creatine kinase (CK), creatinine, urea, glucose, lactate, total bilirubin, γ-glutamyltransferase, total protein, albumin, total calcium, phosphorus, sodium, potassium, chloride, total iron, transferrin (as TIBC) and serum amyloid A (SAA), was carried out on the serum samples using an automated chemistry analyser (Olympus AU 400, Beckman Coulter/Olympus). Serum amyloid A was measured as previously reported (Dondi et al., 2015). Fibrinogen (Siemens), D-dimers (D-DI2 Tina-quant, Roche/Hitachi), and antithrombin (Antithrombin III, Roche/Hitachi) were also measured on plasma samples.

### Histology

Tissue samples, fixed in 10% neutral buffered formalin, were embedded in paraffin within 24–48 h, cut into 5 μm-thick sections and stained with haematoxylin and eosin. The specimen size was standard (1.5 cm in length) and one tissue section from each sample was available for review. Eigth sections from 8 horses were assessed (one colonic tissue section for each horse). Tissue viability was determined by evaluating the morphological changes present in the tissue sections using the scoring system previously described (19, 20) on the basis of the grading of the following parameters: interstitial-crypt ratio (I:C; normal: <1), percentage loss of surface epithelial, percentage loss of the glandular epithelium (superficial and deep), percentage area of hemorrhage (lamina propria and submucosa), percentage area of edema (lamina propria and submucosa) and thrombosis. These criteria were each given a score from 1 to 5, leading to an overall cumulative score/section ranging from 10 to 50 (20). The cut-off between no lesions or lesions of mild severity, and serious necrotic/haemorrhagic injuries was 20 (0–5: no lesions; 6–19: lesions of mild severity, no necrosis; > 19 severe necrotic/haemorrhagic injury) (17).

### Immunohistochemistry

The tissue was fixed for 48 h in 4% paraformaldehyde in 0.1 M PBS, pH 7.4, at 4°C. To obtain frozen tissue, small longitudinal portions (1 × 0.3 cm) of the cranial intestine were washed in PBS and stored at 4°C in PBS containing 30% sucrose and sodium azide (0.1%). On the following day, the tissues were placed in a mixture of PBS−30% sucrose azide and Optimal Cutting Temperature (OCT) compound (Sakura Finetek Europe, NL) at a ratio of 1:1 for an additional 24 h before being embedded in 100% OCT in Cryomold® (Sakura Finetek, Zoeterwoude, NL, Europe). The sections were prepared by freezing tissues in isopentane cooled in liquid nitrogen. Serial longitudinal sections (14–16 μm thickness) of the tissues were cut on a cryostat (Leica, Wetzlar, Germany) and mounted on polysine-coated slides. The sections were stored at −80°C until the histological and/or immunohistochemical experiments were begun. Six sections for each animal were prepared.

### Single Immunofluorescence Experiments

The cryostat sections were rehydrated in PBS and were then processed for immunostaining. To block non-specific binding, the sections were incubated in a solution containing 10% normal goat serum (Colorado Serum Co., Denver, CO, #CS 0922) and 0.5% Triton X-100 (Merck, Darmstadt) in PBS for 1 h at room temperature (RT). Thereafter, the sections were incubated in primary antibody rabbit anti-PAR_2_ (1:100, Santa Cruz Biotechnology, Santa Cruz, Dallas, TX, USA, Cat# sc-5597, RRID:AB_2231455) diluted in antibody diluent (1.8% NaCl in 0.01 M sodium phosphate buffer containing 0.1% sodium azide) for 24 h at +4°C. After washing in PBS (3 × 10 min), the sections were incubated in secondary antibody goat anti-Rabbit (1:400, Alexa Fluor 594, Thermo Fisher Scientific, Waltham, MA, USA, Cat# A-11037, RRID:AB_2534095) diluted in PBS for 1 h at RT. After washing in PBS (3 × 10 min), the slides were coverslipped with buffered glycerol (pH 8.6). Primary and secondary antobody solutions contained normal goat serum (10%) and Triton X-100 (0.5%). All the incubations were carried out in a humid chamber. The anti-PAR_2_ antibody utilized in this study was tested for its specificity by Western blot analysis which indicated that it was specific for the targeted molecules. The omission as well as the replacment of the secondary antibodies with inappropriate secondary antibodies resulted in the elimination of all the immunoistochemical staining.

### Double Immunofluorescence Experiments

The sections used for neuron assessments were incubated for 24 h at room temperature with a primary antibody solution containing rabbit anti-PAR_2_ (1:200, Santa Cruz Biotechnology, Cat# sc-5597, RRID:AB_2231455) and mouse anti-HuC/D (1:100, Molecular Probes, Eugene, OR, USA, Cat# A-21271, RRID:AB_221448). These sections were washed in PBS (3 × 10 min) and were then incubated with a secondary antibody solution containing Alexa Fluor 488-conjugated goat anti-mouse immunoglobulin G (IgG) (1:400, Molecular Probes, Cat# A-11029, RRID:AB_138404) and Alexa Fluor 594-conjugated goat anti-rabbit IgG (1:400, Molecular Probes, Cat# A-11012, RRID:AB_141359) in PBS. The slides were then washed in PBS (3 × 10 min) and were finally coverslipped with buffered glycerol (pH 8.6). The primary and secondary antibody solutions contained normal goat serum (10%) and Triton X-100 (0.5%).

### Analysis of the Sections

The immunofluorescence preparations were observed using a Nikon H550L (Nikon Instruments, Japan) equipped with the appropriate filter cubes for immunofluorescence. The FITC filter for Alexa 488 (Ex 465–495; DM 505; BA 515–555) and the TRITC filter for Alexa 594 (EX 540/25; DM 565; BA 605–655) were used. For the double immunofluorescence analysis, the neurons were first located by the presence of a fluorophore which labeled one antigen; the filter was then switched to a fluorophore specific for a different wavelength to determine whether or not the neuron was labeled for a second antigen. The images were recorded using a Nikon-Qi1Mc photocamera (Nikon Instruments, Japan) and Nikon Elements Version 4.10 software. The contrast and brightness of the figures were adjusted to reflect the appearance of the labeling seen through the microscope using Adobe Photoshop CS3 Extended 10.0 software (Adobe Systems, San Jose, CA).

### RNA Isolation and Quantitative Real Time Polymerase Chain Reaction (RT-qPCR) for PAR_2_

The RNA extraction and qPCR analysis were essentially carried out as reported in a previous study (18). Briefly, samples of scraped pelvic flexure mucosa were pulverized using a mortar and pestle, and liquid nitrogen; 25 mg of each sample were then lysed in Tissue Lyser TL (50 Hz for 5 min, 2 beads diameter 2 mm, Qiagen, Hilden, Germany). The total RNA extraction was carried out using NucleoSpin RNA II (Macherey Nagel, Duren, Germany) instructions. The RNA was spectrophotometrically (Denovix Inc. Wilmington, DE, USA) quantified (A260 nm), and its quality was determined by gel electrophoresis on 1% agarose. Subsequently, 1 μg of RNA was reverse-transcribed to cDNA using an iScript cDNA Synthesis Kit (Bio-Rad Laboratories Inc., Hercules, CA, USA) in a final volume of 20 μl. Real-time qPCR was carried out using a CFX 96 Real Time System (Bio-RAD Laboratories Inc., California, USA) and iTaq Universal SYBR Green Supermix (Bio-RAD Laboratories Inc., California, USA). All the samples were analyzed in duplicate (10 μl/well).

In addition to the RNA samples from the affected gastrointestinal tracts (CVH group), samples of large colon intestine (*n* = 8) collected from the healthy adult horses (HLT group) after slaughter were reanalysed using qPCR as described in a previous paper (17).

The PAR_2_ and reference genes (GAPDH; HPRT, and β- actin) primer sequences, expected PCR product lengths and the accession numbers in the National Center of Biotechnology Information (NCBI) database are shown in Table 1. Primers were designed using Beacon Designer 2.07 Software (Premier Biosoft International, Palo Alto, CA) and they were chosen according the subsequent specification: primers located on different exons, length 16-28 bp, avoid the secondary structure, 3' stability and specificity. The stability of the reference genes were evaluated by the Biogazelle's qbase plus software (include in the CFX 96 Real Time system). Real time efficiency curve for PAR_2_ was evaluated using pooled cDNA derived from healthy and pathological samples starting at 100 ng with 5 subsequent dilutions (20; 4; 0.8; 0.16; 0.032 ng) amplified in triplicate. The obtained curve data were: efficiency 84%, slope−3.776 and *R*^2^ 0.971 including all samples with the largest variation on the most diluted one.

**Table 1:** Forward and reverse primer sequences, polymerase chain reaction (PCR) product lengths and accession numbers (AN) in the NCBI (National Center of Biotechnology Information) database.

**Gene**	**Sequence (5^**′**^-3^**′**^)**	**PCR length (bp)**	**AN**
PAR-2	For.: GGAAGGAGCCTCATTGGTAAG Rev.: ACAAGTGGAAGACAGACAGTAG	140	KF021489
GAPDH	For.: TGGTGAAGGTCGGAGTAAAC Rev.: TGTAGTTGAGGTCAATGAAGGG	120	NM_001163856
HPRT	For.: GCGTCGTGATTAGTGATGATGAAC Rev.: ACAGAGTGCTACAATGTGATGGC	179	AY372182
β-ACTIN	For.: ATCGTGCGTGACATCAAGGA Rev.: AGGAAGGAGGGCTGGAAGAG	169	AF035774.1

The expression level of the PAR_2_ gene was determined using the 2 ^−ΔΔ*Ct*^ method (17) in which ΔCt = (Ct _PAR2_-Ct _mean ref.genes_) and ΔΔCt = ΔCt _(CVH tract)_ - ΔCt _(HLTtract)_.

### PAR_2_ Western Blot Analysis

Protein extraction and Western blot analysis were essentially carried out as previously reported (18).

Aliquots containing 20 μg of proteins were separated on Bolt 4-12% bis-Tris Plus^n^ (Thermo Fisher Scientific, Rockford, IL, USA) for 55 min at 165 V. The proteins were then electrophotoretically transferred onto a nitrocellulose membrane. The membranes were then incubated overnight at 4°C with a 1:500 dilution of the primary antibody, rabbit anti-PAR_2_ (Santa Cruz Biotechnology, Cat# sc-5597, RRID:AB_2231455), in Tris Buffered Saline-T20 (TBS-T20 20 mM: Tris-HCl, pH 7.4, 500 mM NaCl, 0.1% T-20) with 2% milk powder. After several washings with PBS-T20, the membranes were incubated with the secondary biotin-conjugate antibody (1:100.000 dilution in TBS-T20, 1 h at RT) and then with a 1:1000 dilution of an anti-biotin horseradish peroxidase (HRP)-linked antibody (1 h at RT). The intensity of the chemiluminescent signal of the resultant bands was acquired using the Chemidoc Instrument (Bio-Rad) and the bands were measured using LabImage Software (Bio-Rad).

In order to normalize the PAR_2_ data on the reference protein, the membranes were stripped (briefly: the membranes were washed for 5 min in water, then for 5 min in 0.2 M NaOH and were then washed again in water) and reprobed with the reference α-tubulin (1:500; cod.TU-01 Thermo Scientific Waltham, MA, USA). The relative PAR_2_ protein content was normalized to the value of the reference protein and was expressed as arbitrary units (AUs) for each healthy and pathological tract. The Western blots were developed using a chemiluminescent substrate (Clarity ECL Western Blot Substrate, Bio Rad) according to the manufacturer's instructions. The intensity of the luminescent signal of the resultant bands was acquired by Chemi Doc Instrument (Bio-Rad) using LabImage Software (Bio-Rad). The relative PAR_2_ protein content was normalized on the mean value of the reference protein and was expressed as AUs.

In addition to the samples derived from displaced colon, the proteins of the small intestines collected from the healthy adult horses after slaughter and described in a previous paper (18) were analyzed.

### RT^2^ Cytokine Array

To evaluate the expression of the key molecules involved in the inflammatory response, a focused panel of equine cytokine and chemokine genes (RT^2^ Profiler™ PCR Array Horse Cytokines & Chemokines, Cat. No. 330231, PAEC-011ZA, Qiagen, Hilden, Germany) was used.

Total RNA from the healthy or the pathological tracts of the large colon was purified and quantified as previously mentioned above. Subsequently, one microgram of RNA pooled from a healthy or a pathological animal was retro-transcribed, using an RT^2^ First Strand Kit (Qiagen, Hilden, Germany) following the manufacturer's instructions, in a 20 μl final volume to obtain the cDNA. RT^2^ Profiler™ PCR Array Horse Cytokines & Chemokines was carried out according to the manufacturer's instructions, using CFX 96 Touch (Bio-Rad Laboratories, Hercules, CA).

In order to validate the Array results, real time qPCR was carried out for those genes upregulated more than 10 times (IL-8; MIP2-Beta; CXCL1) with respect to the healthy sample. A master mix of the following reaction components was prepared in a 25 μl final volume using RT^2^ SYBR green master mix and RT2 Primer Assay (RT^2^ qPCR Primer Assay for Horse IL8, MIP2-beta; CXCL1, Cat. No. PPE00415B, PPE02633A; PPE00308; respectively, Qiagen) according to the manufacturer's instructions. One microliter of cDNA was added to 24 μl of the master mix. All the samples were analyzed in duplicate. The qPCR protocol used was: 10 min at 95°C, 40 cycles at 95°C for 15 s and at 60°C for 30 s, followed by a melting step from 55°C to 95°C (80 cycles of 0.5°C increase/cycle). The gene expression was evaluated using the ΔCt method (mean reference gene Ct _GAPDH;HPRT, β−*Actin*_-interest gene Ct). The relative messenger RNA (mRNA) expression of the genes tested was calculated in relation to the control cells using the 2^−ΔΔ*ct*^ method (21).

### Statistical Analysis

The clinicopathological data were reported as median and range (minimum-maximum). The mRNA and protein data of the HLT and the CVH groups were evaluated using student's *t*-test (*p* < 0.05) (GraphPad Prism V.5.01, GraphPad Software, La Jolla, CA, USA).

## Results

### Animals

Eight horses (5 females, 2 geldings and one stallion) having a mean age of 15 ± 6 years met the inclusion criteria and were enrolled in the study (CVH group). All the patients underwent an exploratory laparotomy, and a diagnosis of large colon volvolus was confirmed.

During the surgical procedure, a pelvic flexure colotomy was performed to remove all the fecal material, and repositioning of the large colon was subsequently carried out.

The postoperative period of all the patients was followed at the VUH. The horses received the specific therapy for postoperative large colon colic horses: fluid therapy (Ringer lactate 4–10 ml/kg/h), antibiotic therapy (ampicillin 20 mg/kg IV every 8 h; gentamicin 6.6 mg/kg every 24 h) and a prokinetic (intrastigmine 0.02 mg/kg every 2 h until normal motility of the large colon was restored), antiflammatory drugs (flunixine meglumine 1 mg/kg IV twice a day) and an anticoagulant (calcium heparin 5000 UI/kg SC). Within 48 h after the surgical procedure, three horses developed severe abdominal pain with an increased heart rate (range 79-110 beat for minutes), and they were euthanised the following 24 h. Five out of the 8 horses were discharged 7–10 days after admission.

### Clinicopathological Evaluation

The clinical pathological data are summarized in Table 2. An increase in CK, D-dimer and SAA concentrations was observed. No other hematological or biochemical alterations were recorded.

**Table 2:** Clinicopathological values of the horses included in the study at admission.

**Variable**	**Unit**	**Result**	**Reference interval**
**Hematology**			
Haematocrit value	%	41.8 ± 15.1	31.5–50
Hemoglobin	g%	13.5 ± 3.40	11.0–19.0
RBCs	10^3^ × Cells/mm^3^	8,753 ± 3,181	5,500–12,500
WBCs	Cells/mm^3^	7,316 ± 2,831	5,000–13,000
Platelets	Cells/mm^3^	104,375 ± 35,633	100,000–600,000
**Chemistry**			
AST	U/L	219 (123–1626)	60–280
CK	U/L	703 (47–1743)	90–270
LDH	U/L	756 (338–2196)	240–970
Glucose	mg/dL	145 (105–177)	80–120
Urea	mg/dL	39 (33–78)	15–40
Creatinine	mg/dL	1.35 (1.3–3.69)	0.90–1.50
Phosphate	mg/dL	3.8 (2.5–5.27)	1.75–4.90
Total calcium	mg/dL	9.25 (6.2–10.6)	11.3–13.5
Sodium	mEq/L	138 (132–154)	135–143
Potassium	mEq/L	3.5 (3.2–4.2)	2.8–4.5
Chloride	mEq/L	97 (90–108)	98–104
Magnesium	mg/dL	1.33 ± 0.17	1–2.6
Albumin	g/dL	2.53 (1.37–2.92)	2.90–3.70
Total protein	g/dL	4.86 (3.17–5.61)	5.90–7.30
A:G	g:g	1.05 (0.76–1.32)	0.7–1.3
Total bilirubin	mg/dL	2.53 (0.55–6.86)	0.90–2.60
GGT	U/L	8.7 (2.1–116)	3.6–20.6
Total iron	μg/dL	77 ± 46	55–260
TIBC	μg/dL	300 ± 96	305–420
TIBC saturation	%	27 ± 14	12–70
SAA	μg/mL	94 ± 138	0–10
**Coagulation**			
Antithrombin	%	134 (68–191)	140–230
D-dimer	μg/dL	2.28 (0.14–3.2)	0.20–0.48
Fibrinogen	g/L	3.43 (1.58–5.07)	1.60–4.10

*Data are reported as mean±standard deviation or median and (minimum-maximum), based on their distribution*.

*RBCs, red blood cells; WBCs, white blood cells; AST, aspartate-aminotransferase; CK, Creatine-kinase; LDH, Lactate-dehydrogenase; A:G, Albumin to globulin ratio; GGT, Gamma-glutamyl-transferase; TIBC, Total iron binding capacity; SAA, Serum amyloid A*.

### Histology

**Figure 1:**
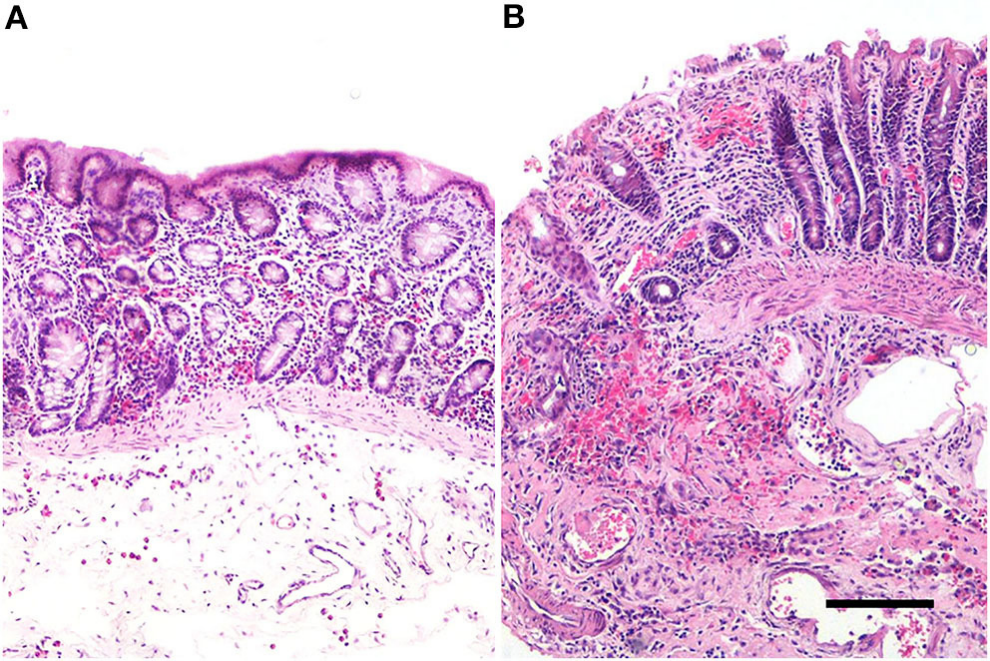
Equine pelvic flexure Histology. Representative sections of two cases of different degree of severity. **(A)** Mucosal and submucosal lesions of mild severity, represented by mild mucosal hemorrhages and moderate submucosal oedema, in association to a mild inflammatory lymphoplasmacytic infiltrate; **(B)** mucosal and submucosal lesions of severe injury. In the mucosa, the luminal epithelium show a severe and diffuse superficial loss, multifocal haemorrages and a severe lymphoplasmacytic infiltrate. In the submucosa is also observed a severe hemorrhage and a moderate chronic inflammatory infiltrate. Haematoxylin & Eosin (H&E); scale bar, 200 μm.

**Figure 2:**
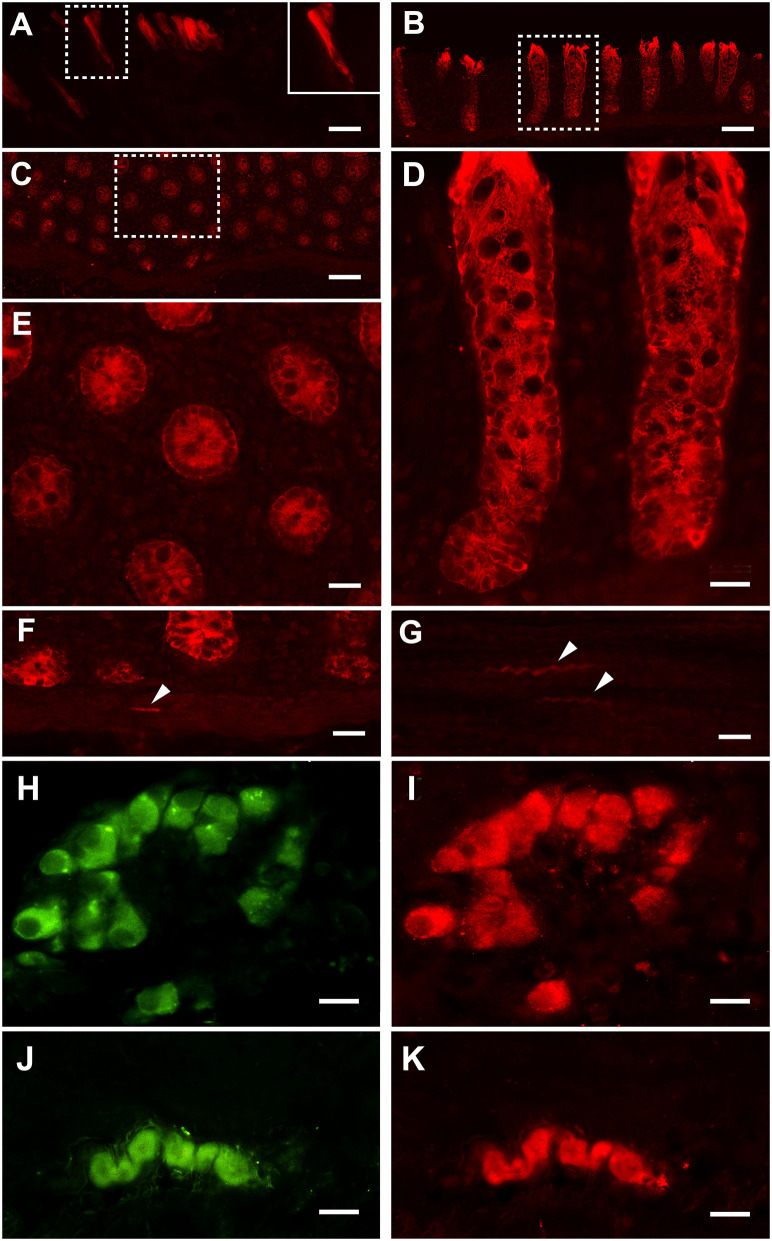
Proteinase-activated receptor 2 immunostaining (PAR_2_-IR) in the large colon. Note the strong immunoreactivity in the enterocytes **(A)**. Inset in **(A)**: high magnification of the boxed area. Strong immunoreactivity for the PAR_2_ is located in the intestinal glands (**B**: longitudinal section; **C**: transverse section). **(D)** High-magnification view of the boxed in area **(B)**. **(E)** High-magnification view of the boxed in area **(C)**. Smooth muscle cells (arrowheads) distributed in the *muscolaris mucosae* **(F)** and the muscular layer **(G)** occasionally show weak immunoreactivity. In the submucosal **(H,I)** and myenteric **(J,K)** plexi, neurons immunoreactive for the HuC/D (in green: **H,J**) are immunoreactive for PAR_2_ (in red: **I,K**). Scale bars: **(A,D,E,F,G)** = 25 μm; **(B,C)** = 100 μm; **(H–K)** = 25 μm (scale bar for insets = 10 μm).

In the colonic samples of the eight horses evaluated, the score ranged from 11 to 20; 6 samples were not necrotic, having lesions of mild severity (ranging from 11 to 15–Figure 1A) and 2 sections were necrotic (score equal to 20-Figure 1B). The histomorphological score system data are summarized in Table 3. The inflammatory infiltrate was mainly chronically moderate diffuse lymphoplasmcellular (in 6 out of 8 sections) and, in two samples, there was also the compresence of a small number of neutrophils and a moderate number of eosinophilic granulocytes. In the remaining 2 samples, however, the infiltrate was represented almost exclusively by large numbers of neutrophils and eosinophilic granulocytes.

**Table 3:** Results of the histomorphologic scoring system evaluation of the tissue sections included in the study.

**Horse**	**Interstitial- crypt (I:C) ratio (normal: <1)**	Surface epithelium loss^*^	Glandular epithelium loss^*^		Areas of hemorrhages^*^		Areas of oedema^*^		**Thrombosis**^****^°^****^		**Total Score**
			**S**	**D**	**LP**	**SM**	**LP**	**SM**	**LP**	**SM**	
CVH1	3	4	3	2	1	2	2	1	1	1	20
CVH2	1	2	1	1	2	2	2	2	1	1	15
CVH3	2	1	1	1	1	2	1	2	1	1	13
CVH4	2	1	1	1	2	1	1	2	1	2	14
CVH5	2	2	1	1	3	3	2	2	1	3	20
CVH6	1	1	1	1	1	1	1	2	1	1	11
CVH7	2	1	1	1	1	2	1	1	1	1	12
CVH8	1	1	1	1	1	2	1	1	1	1	11

*S, superficial; D, deep; LP, lamina propria; SM, submucosa. CVH, colon volvulus horse*;

* *score 1 (0–20%), score 2 (21–40%), score 3 (41–60%); score 4 (61–80%); score 5 (81–100%)*.

° *Number of thrombi: score 1 (0); score 2 (1); score 3 (2), score 4 (3); score 5 (>4)*.

### Immunofluorescence Experiments

Strong PAR_2_ immunostaining was observed in the enterocytes (Figure 2A) and the intestinal glands (Figures 2B–E). Weak immunostaining could also occasionally be observed in the smooth muscle cells located in the *muscularis mucosae* (Figure 2F) and muscular layer (Figure 2G); PAR_2_ immunoreactivity was also observed in many neurons of the submucosal (Figures 2H,I) and the myenteric (Figures 2J,K) plexi.

### RNA Isolation and RT- qPCR for PAR_2_

**Figure 3:**
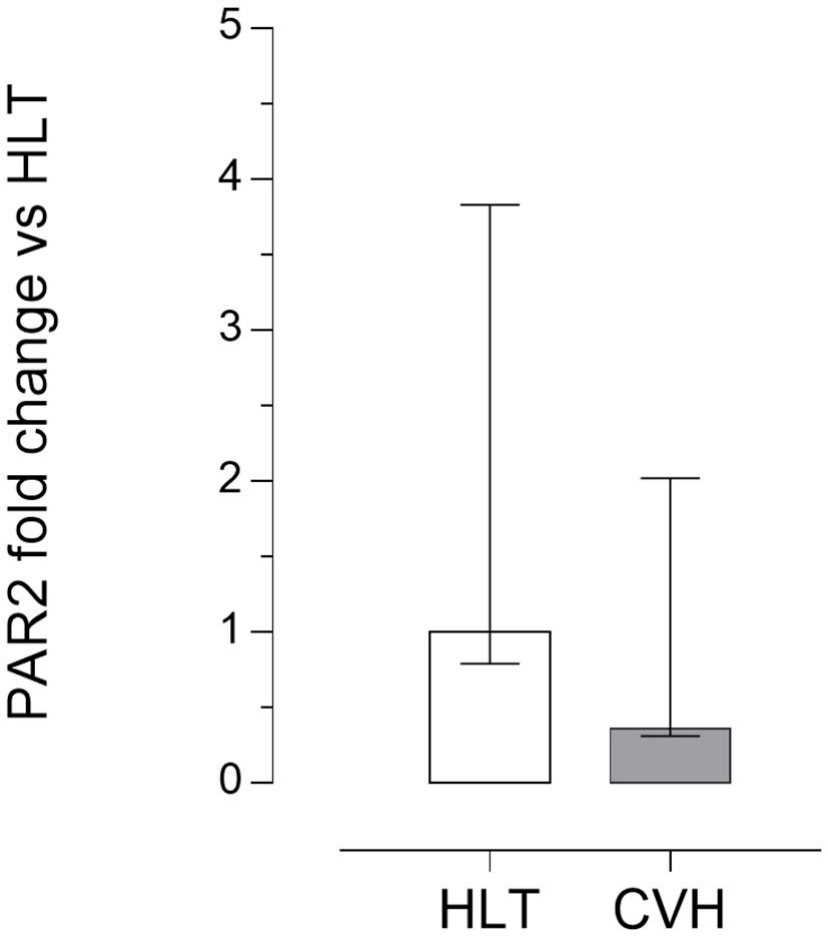
Quantitative Real time PCR for the PAR_2_ in the pelvic flexure specimens. The gene expression level of PAR_2_ during colon volvulus (CVH group, *n* = 8) was calculated as fold of change (2^−ΔΔ*Ct*^ method) in relation to the colon tracts isolated from the healthy horses (HLT group, *n* = 8). Data represent the mean ± the range of relative expression of eight biological replicates per group; each experiment was repeated twice. No statistically significant differences in gene expression were observed (student *t*-test, *p* < 0.05).

Quantitative PCR data demonstrated that PAR_2_ mRNA was detectable in all of the samples analyzed. No significant statistical difference of its expression in the pelvic flexure tracts was observed between the healthy (HLT) and the pathological (CVH) groups (Figure 3).

### PAR_2_ Western Blot Analysis

**Figure 4:**
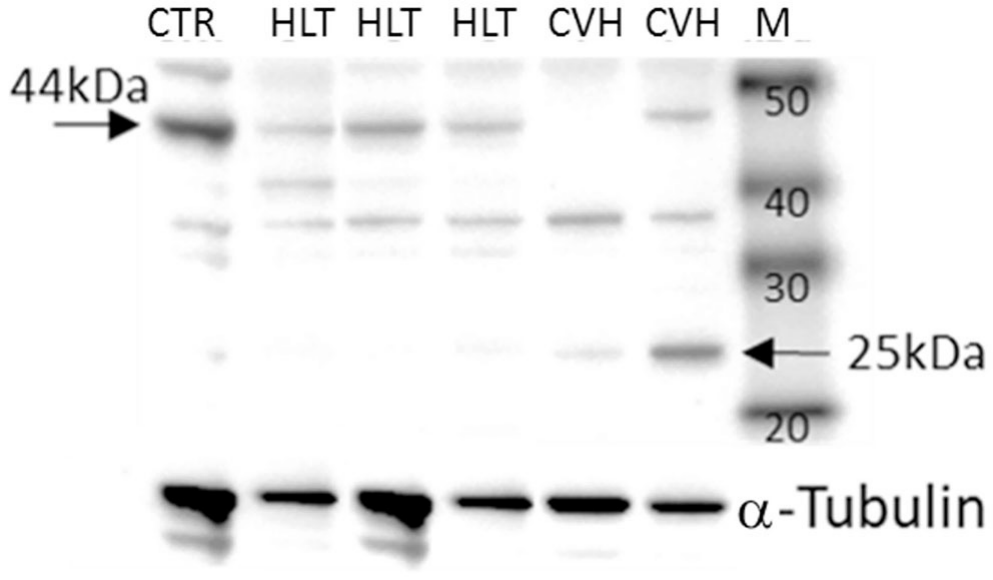
Representative image of Western blots of PAR_2_ proteins and the reference proteins, α-tubulin, in the horses during colon volvulus (CVH group) and in the colons of the healthy horses (HLT group). CTR: positive control for PAR_2_ (healthy jejunum tract); M: Molecular weight marker (kDa).

Under denaturing gel electrophoresis conditions (SDS–PAGE), western blots of the mucosa of the colon tracts showed a clear band of the expected molecular weight for PAR_2_ (~44 kDa) and a smaller band (about 25kDa), suggesting an internalization and degradation of the receptor after its activation (17) (Figure 4).

**Figure 5:**
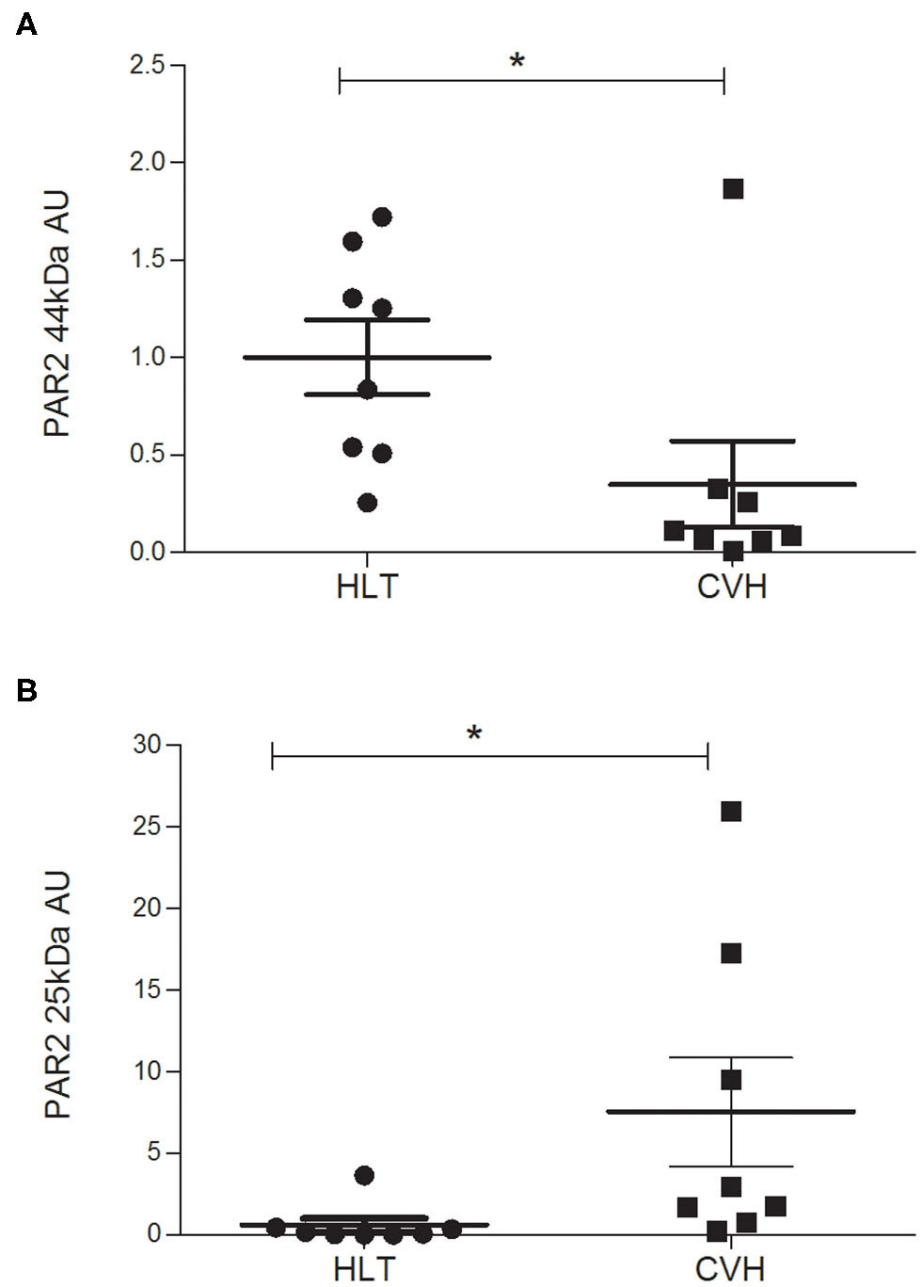
Protein expression level of the PAR_2_ in horses during colon volvulus (CVH group, *n* = 8) and in colon of healthy horses (HLT group, *n* = 8). The densitometric analyses were carried out on the expected molecular weight for the PAR_2_ (~44 kDa, **A**) as well as on the smaller band (~25 kDa, **B**). The *indicates the statistically significant differences between the HLT and the CVH groups (student *t*-test, *p* < 0.05). AUs: arbitrary units in relation to reference protein (α-tubulin).

The protein quantification of PAR_2_ showed a statistically significant reduction of the receptor (band of 44kDa) together with a statistically significant increase in the amount of the smaller band (25kDa) in relation to control tissue (Figure 5) in agreement with the mechanism of activation, internalization and degradation of the receptor.

### RT^2^ Cytokine Array

**Figure 6:**
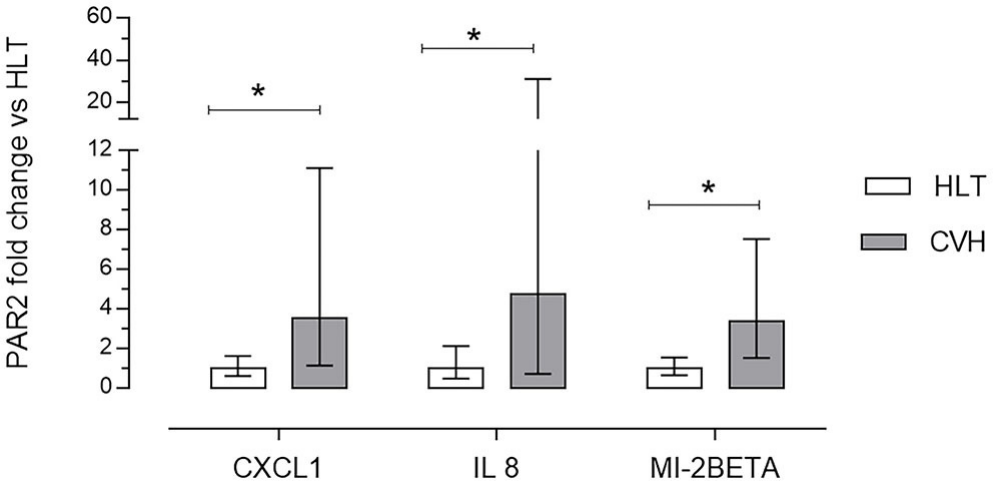
Array validation using quantitative real-time polymerase chain reaction (RT-qPCR) of C-X-C motif ligand 1 (CXCL1), interleukin 8 (IL8) and macrophage inflammatory protein 2 beta (MI-2BETA) in the colon mucosa of the healthy horses (HLT group, *n* = 8) or in the colon mucosa of the horses with volvulus (CVH group, *n* = 8). The relative mRNA expression (fold of change) was calculated in relation to the HLT sample using the 2^−ΔΔ*ct*^ method. Data represent the mean ± the range of relative expression of eight biological replicates; each experiment was repeated twice. The data were analyzed using the Student's *t*-test. Significant differences are indicated by *p < 0.05.

Large Colon volvulus showed an altered gene expression profile; of the 84 genes of the RT^2^ Profiler™ PCR Array Horse Cytokines & Chemokines, 33 genes were upregulated (fold of change >1), 21 genes were downregulated (fold of change <1) and 29 were undetectable (ND) (Table 4). Three genes (CXCL1; IL8, MIP-2BETA) were upregulated more than 10-fold (Table 4) and the array on these three genes was verified. The array validation confirmed the gene expression profile determined by the array analysis (Figure 6); the Authors observed a significant increase in their expression in the pathological samples in relation to the healthy colons.

**Table 4:** Transcriptional profile of the cytokines and chemokines in the large colon of the horses with large colon displacement and volvulus; 84 genes of the RT^2^ Profiler™ PCR Array Horse Cytokines & Chemokines were reported.

**Gene**	**Fold change**	**Gene**	**Fold change**	**Gene**	**Fold change**	**Gene**	**Fold change**
BMP2	1.22	IL15	1.33	MIF	1.27	OSM	ND
CCL11	1.81	IL16	0.71	CD40LG	1.02	TNFSF10	0.80
CCL13	3.50	IL17A	ND	FLT3LG	0.80	CD70	1.04
CCL2	5.72	IL18	1.34	IL24	ND	C5	1.75
CCL3	ND	IL18R1	ND	CCL4	1.63	CX3CR1	ND
CCL5	0.64	IL1A	3.22	LTA	ND	IL17F	ND
CCL8	ND	IL1B	4.98	LTB	0.82	IL2RB	0.46
CCR2	ND	IL1R1	2.46	IL7R	0.70	IL10RA	ND
CCR5	1.01	IL1RN	2.12	AIMP2	ND	CSF1	0.80
CSF3	ND	IL23A	0.90	NAMPT	1.48	LIF	0.72
CXCL1	15.10	IL4	0.56	IL33	0.45	CXCL11	0.56
CXCL10	1.69	IL4R	1.23	IL5RA	0.83	CCL20	0.85
CXCL2	6.76	IL5	ND	TNFSF11	ND	CXCL13	0.89
CXCL6	5.80	IL6	ND	TNFSF14	0.51	IL3	ND
CXCL9	1.23	IL6ST	3.20	IL17B	ND	PF4	ND
FASLG	0.86	IL7R	1.50	TNFSF4	ND	MIP-2BETA	11.05
GM-CSF	0.67	IL8	161.57	CCL24	1.23	TNF	0.80
IFNG	0.21	IL9R	ND	CCL22	ND	TNFRSF11B	ND
IL10	0.53	TGFB2	2.56	IL9	ND	TNFSF13	1.11
IL12B	ND	IL17C	ND	EBI3	ND	TNFSF13B	1.62
IL-13	ND	IL10RB	1.08	IL21	ND	VEGFA	1.03

*The relative messenger RNA (mRNA) expression (fold of change) of the tested genes was calculated in relation to the large colons of the healthy horses using the 2^−ΔΔct^ method*.

## Discussion

In this paper, the Authors reported the distribution of the PAR_2_ at the level of the pelvic flexure in horses with volvolus of the large colon. In all the patients, the diagnosis was confirmed at explorative laparotomy; the histological evaluation allowed grading the severity of the colon lesions. Three out of the eight patients were euthanised due to clinical deterioration, and the survival outcome was 62.5%. Colon volvulus in horses is a frequent disease, causing colic syndrome; the short- and long-term survival outcomes vary from 35 to 57% (2, 22). The outcome is correlated primarily with the duration of the disease (23) and, after surgery, with the complications related to ischemia/reperfusion injury.

During colon volvulus, the damage to the vessels could result in ischemia. Later on, when the repositioning of the large colon occurs, reperfusion and re-oxygenation exacerbate the tissue injury (4, 24). In particular, ischemia and reperfusion induce the activation of an inflammatory response mediated by the migration of white blood cells into the lamina propria of the large intestine (24).

Of the clinicopathological parameters evaluated in the present cohort, an increase in SAA was recorded, confirming a severe inflammatory pathway, as has previously been described in colic horses (25, 26). The inflammatory activity was also confirmed in bioptic samples of the colon by the significant increase in the expression of cytokines, in particular of IL-8 and CXCL1, as has already been reported in human patients with chronic bowel inflammation (27, 28).

Proteinase activated receptor 2 modulates acute and chronic inflammatory processes in the gastrointestinal tract in both humans and animal models. In these situations, the PAR_2_ in the gastrointestinal tract acts as a pro-inflammatory mediator (29–31), or promotes cellular healing and maintains gastrointestinal motility (30, 32). These effects of PAR_2_ depend on the type of cells involved and are mediated by endogenous or exogenous proteases, but also by cytokine production and by enteric nervous system activation (16, 30, 33–36).

In the present study, PAR_2_ expression was identified in the enterocytes, in the intestinal glands, in the muscular layers of the *muscolaris mucosae*, in the enteric nervous system neurons and in the muscular layers. This same expression had previously been observed in the bowels of healthy horses and in the jejunums of colic horses (17, 18). In the present study the PAR_2_ mRNA expression in the samples collected from the colic horses did not differ significantly from that obtained in the samples collected from the healthy animals. Similarly, in the jejunums of horses with epiploic hernias, the PAR_2_ mRNA expression in the central injured tract, which was the intestinal tract characterized by severe histological lesions, did not differ significantly from that of the non-pathological jejunum samples (17). In previous studies, in animal models of ischemia and reperfusion injury, the PAR_2_ expression in the intestine did not differ if reperfusion occurred or not after the induced ischemic damage (30); in another study the PAR_2_ expression increased in a time dependent manner after intestinal injury and after 60 min of reperfusion (35). These differences could be explained by the different study designs. Yoshida and colleagues (35) simulated the ischemic damage by clamping both the superior mesenteric artery and the celiac trunk for 30 min. In the present study, the Authors included horses with colon volvulus, as a model of spontaneous inflammatory injury of the large intestine. All these horses were referred to the VUH within 6 h from the presentation of the clinical signs, but it was impossible to clearly define the exact time of the onset of the ischemia.

The protein quantification of PAR_2_ in the pathologic colon demonstrated a reduction in the amount of PAR_2_ protein (44kDa) and an increase in the amount of a light protein (25kDa) when compared with samples from healthy colon tracts. The same results were observed in small intestinal tracts following herniation through the epiploic foramen in which the PAR_2_ protein content was lower when compared with the samples of small intestines obtained from the control animals (17) and support the activation of PAR_2_. In fact, PAR_2_ activation consists of the cleavage of the extracellular N-terminal domain by the agonist proteinase. This results in the reduction of the protein level of the receptor and in the formation of an N-terminal peptide of a lower molecular weight. This peptide combines with the extracellular domain (tethered ligand) of the receptor itself, thereby activating the intracellular pathways (37).

Proteinase-activated receptor 2 activation is involved in the pathophysiology of gastrointestinal disease in several ways; of these, it modulates the tryptase induced IL-8 production by eosinophils (38), epithelial cells (36, 39), neutrophils (40) and endothelial cells (41). Even if in the present study, the interleukin-containing cells were not evaluated by immunohistochemistry, an involvement in PAR_2_ mediated interleukin production in the equine colon could be hypothesized as has previously been described in human colonic samples (28). *In vitro* studies showed that IL-8 production increased dose-dependently when PAR_2_ was activated while it was inhibited by a PAR_2_ antagonist (42–44).

CXCL1, IL-8 and MIP-2BETA are chemo-attractants for neutrophils (45, 46); MIP-2BETA is secreted by the epithelial cells during the inflammatory process of the intestinal tract (47). Moreover, the expressions of CXCL1 and IL-8 are reciprocally related; according to the data observed in the spontaneous equine acute models in the present study, both also increase in human patients during ulcerative colitis, a chronic inflammatory disease (28, 48). On the contrary, in PAR_2_ deficient mice, in which intestinal inflammation was experimentally induced with *Toxoplasma gondii* infection, the expression of CXCL1 was lower and the neutrophil infiltration was reduced when compared with wild type littermates (49).

In this study, the most important cellular infiltrates were represented by moderate-diffuse lympho-plasmacellular infiltrates and in only two intestinal section was the infiltrate represented almost exclusively by a large number of neutrophils. Neutrophils were observed in the sections of the two horses with the highest histological score. These cells play an important role in the mechanism leading to the mucosal damage associated with ischemia/reperfusion injury (50). This is related not only to the release of reactive oxygen metabolites and proteases by the neutrophils, but also to the physical alterations produced by their infiltration across the epithelium (50, 51).

The histological differences between ischemia and reperfusion in the equine colon were not evaluated in the present study; however, previous researchers have described the effects of experimentally induced colon ischemia and reperfusion in horses (4, 24). In detail, in the equine colon, the number of neutrophils in the mucosa increased after a brief period of ischemia, with progression after 1 h of reperfusion and peak infiltration after 2 h of reperfusion (4). However, in this latter study, despite the significant infiltration of neutrophils, epithelial repair was histologically evident after reperfusion (4). Therefore, Grosche et al. (4), observed that neutrophils might also play a role in the intestinal mucosa repair process when the ischemic damage is mild. In the present study, the neutrophil infiltration seemed to be related to severe damage of the tissue induced by the ischemic process and associated with a high histological score.

The samples obtained from the horses with the highest histological scores were also characterized by a large number of eosinophils. In previous studies, experimentally induced ischemia or naturally occurring colon volvulus did not change the total number of eosinophils; however, they appeared activated, and they migrated toward the luminal surface of the epithelium (4, 52, 53). These previous papers suggest that eosinophils also play a role in the ischemia and reperfusion mechanisms also in our cohort.

The inflammatory processes in the gastrointestinal tract are also modulated by the enteric nervous system by means of PAR_2_ (29) which is involved in neuronal signaling (54, 55). In fact, when the proteases activate PAR_2_ on the enteric neurons, they release neuropeptides resulting in a neurogenic inflammation characterized by vasodilation, edema and granulocyte infiltration (33). In the present study, immunostaining for PAR_2_ was observed in the myenteric and the submucosal plexi neurons, as has previously been observed in healthy horses (18). These results therefore additionally support the role of PAR_2_ in mediating ischemia/reperfusion injury in the equine colon.

The contribution of PAR_2_ in regulating the mechanisms involved in ischemia/reperfusion injury are still to be completely elucidated. However, experimental studies in animal models have demonstrated that the administration of a PAR_2_ antagonist attenuated the inflammation related to tissue damage (35, 56). In fact, in a rat model of reperfusion injury, when an anti-rat PAR_2_ cleavage site antibody was administered intraperitoneally before the ischemia, smaller erosions of the intestinal epithelial surface, fewer neutrophils and overall less extended intestinal damage was observed as compared with the untreated animals (35). Similarly, in a rat model of pancreatitis, anti-rat PAR_2_ cleavage site antibody administration determined the inhibition of cytokine production (56). Therefore, PAR_2_ should be taken into consideration in clinical activity, as a new therapeutic target, to modulate inflammatory changes related to ischemic colonic lesions and, maybe, to reduce the risk of postoperative complications such as ischemia/reperfusion injury.

### Conclusions

In clinical practice, the manual repositioning of the displaced large colon is the resolving treatment for colon volvulus. However, the decision of the surgeon to remove the compromised tract still depends on the gross aspect of the mucosa.

In the present study the activation of the PAR_2_ in equine intestine with colon volvolus, as related with the tissue damage has been demonstrated. In addition, since in experimental model the administration of a PAR_2_ antagonist has been described as a promising therapy for modulation of inflammatory processes, further studies are warrented to evaluate if the administration of a PAR_2_ antagonist could be beneficial also in equine patients for the prevention of inflammatory-mediated intestinal damage secondary to ischemia or ischemia-reperfusion injury.

The limitation of the present study was the small number of animals included; however, the specific inclusion criteria allowed the Authors to include equines with comparable disease, especially in the time of onset of the large colon disease.

## Data Availability Statement

The raw data supporting the conclusions of this article will be made available by the authors, without undue reservation, to any qualified researcher.

## Ethics Statement

The animal study was reviewed and approved by Ethical Scientific Committee for Experimental Animals of the University of Bologna (Prot. n 15-IX/, 08/ 05/2012). Written informed consent was obtained from the owners for the participation of their animals in this study.

## Author Contributions

CL, CBo, AZ, CBe, FD, MM, and MF carried out data management, analysis, and interpretation of the results. RR, AS, and NR carried out the sample collection. NR and CL wrote the first draft of the manuscript. CBo and AZ wrote sections of the manuscript. NR and AZ contributed to the conception and design of the study. All authors contributed to the manuscript revision and approved the version submitted.

## Conflict of Interest

The authors declare that the research was conducted in the absence of any commercial or financial relationships that could be construed as a potential conflict of interest.

## Abbreviations

CVH: colon volvulus horse
HLT: healthy horses
IBD: inflammatory bowel diseases
PAR: proteinase-activated receptor
PBS: phosphate buffered saline
qPCR: real time polymerase chain reaction
RT: room temperature
VUH: Veterinary University Hospital.
